# ‘A roller coaster of emotions’: a phenomenological study on medical students lived experiences of emotions in complex simulation

**DOI:** 10.1186/s41077-021-00177-x

**Published:** 2021-07-03

**Authors:** Claudia C. Behrens, Erik W. Driessen, Diana H. Dolmans, Gerard J. Gormley

**Affiliations:** 1 Medical Education Unit, Universidad Católica del Norte, Coquimbo, Larrondo 1281, Coquimbo, Chile; 2 School of Health Professions Education, Maastricht University, Universiteitssingel 60, 6229 ER Maastricht, The Netherlands; 3 Centre for Medical Education, Queen’s University Belfast, University Road Belfast, BT7 1NN Belfast, Northern Ireland

**Keywords:** Emotions, Medical students, Simulation, Learning

## Abstract

**Background:**

Simulation-based education can induce intense learner emotions. The interplay between emotions and learning is less well understood. Gaining greater insights into learner emotions has potential to guide how best we manage emotions and optimise learning. This study aimed to understand learners’ lived emotional experiences in complex simulation and the perceived impact on learning.

**Methods:**

Eight final-year medical students participated in the study. Wearing video-glasses, participants took part in a ward-based simulation. Video-footage was used to elicitate exploratory interviews and analysed using Template Analysis reflexively.

**Results:**

Analysis yielded four main themes: ‘nervous anticipation’: encapsulating the fear, anxiety and uncertainty experienced by learners prior to simulation; ‘shock and awe’: feelings of anxiousness and being overwhelmed at the start of a simulation; ‘in the moment: flowing or buffeting with the emotions’: experiencing fear of being judged as incompetent, but also experiencing positive emotions such as satisfaction; ‘safe-landing?’: whilst debriefing aimed to encourage more positive emotions, negative emotions about the simulation could persist even with debriefing.

**Conclusions:**

Complex simulation can evoke intense emotions in students. If students experienced a positive progression, they reported positive emotions and felt competent which was perceived to have a positive impact on learning. If students experienced failure, they reported strong negative emotions which made them question about their future performance and was perceived as negative for learning. Bringing to the surface these complex emotional dynamics, could permit educators to be aware of and adapt the *emotional climate* within simulation in order to optimise learning.

## Introduction

Simulation is a well-established modality of training in health profession education [1–5]. By mixing with other teaching methods, simulation is used for developing medical student’s clinical skills and preparation for practice [6, 7]. Simulation can provide safe and guided learning opportunities to advance students skills, particularly in the management of highly time-dependent and challenging clinical events such as cardiac arrest. At the same time, simulation can be highly emotive for learners; not only because it extends medical students beyond their *comfort zone*, but also due to the nature and contexts of such high-acuity scenarios [8, 9]. Whilst such emotions have potential to positively enhance learning, they also have the capacity to hinder learning [10].

With increasing challenges in our clinical working environments, there has been a greater utilization of more complex simulations. Examples of this include *ward-based simulations* (WRS) [7, 11, 12]. Presenting learners with greater challenges within simulation can induce intense emotions in learners, without a clear understanding of their impact on their learning. Furthermore, there are inherent risks of having intense negative emotional experiences and their capacity to cause psychological distress [13–16].

Emotions have been defined as ‘acute, intense, and typically brief psycho-physiological change that results from a response to a meaningful situation in an individual’s environment’ [17]. Emotional states can not only influence what students learn, but also how students transfer their learning into new situations [18]. For example, negative emotions can impair working memory, immediate recall and clinical reasoning [19]. Equally, more positive emotional states can promote student learning by increasing motivation, creativity and the use of deeper processing strategies to solve problems [10, 20]. The control value theory of achievement emotions can help to understand the role of emotions in education. Achievement emotions are defined as “emotions tied directly to achievement activities or achievement outcomes” [20]. Control value theory, groups achieve emotions by their valence (positive vs. negative), degree of activation (activating vs. deactivating) and object focus (activity vs. outcome). Using these dimensions, the theory proposes a three-dimensional taxonomy of achievement emotions (valance, activation and object). The control value theory explains the relation between achievement emotions and cognitive processes, as well as motivation. As an example, boredom experienced during a dull simulation session would be considered a negative, deactivating, activity-related achievement emotion; whereas the pride associated with arriving at a correct diagnosis with a challenging patient presentation would be considered a positive, activating, outcome-related achievement emotion. This theory points out that deactivating emotions may be more detrimental for learning due to the tendency to encourage disconnection from the learning activity [17]. However, some negative emotions, such as shame, could also promote learning increasing internal motivation and, in some cases, memory consolidation [18].

As simulation-based education continues to evolve and become more complex, there is a need to gain a greater understanding of learner emotional states that occur during such simulations. The literature on simulation-based education reports mixed conclusions about learners’ emotional states and their interplay between learning and performance. Research conducted by Fraser et al. focused on learner emotions and cognitive load. They showed that emotions can increase extraneous cognitive load and reduce learning outcomes [21, 22]. Other studies demonstrated that physical environments in more complex simulation can be a source of stress and distraction, thereby generating greater extraneous cognitive load and potentially hamper learning [23]. In a recent study, Behrens et al. [10] explored the effect of *achievement emotions* in learning when simulating. They found that in complex ward-based simulations, positive achievement emotions such as pride and enjoyment can be evoked in medical students and appear to support their learning.

Simulation-based education is a constructed form of teaching and under the control of faculty staff. Gaining greater insights to the emotions experienced by learners in complex simulations has the potential of providing clearer lines of action of how to construct simulation in order to optimise learning. However, a challenge in exploring learner’s emotions in simulation is the ability to capture a true sense of their emotions as they appear *in-the-moment.* Capturing the nuances of such emotional experiences can be difficult for individuals to express, i.e. tacit knowledge. Often, learners’ emotional experiences are explored post hoc and therefore subject to recall bias and learners’ desire to convey a different presentation of their self to others [24]. By unearthing such lived experiences of learners’ emotions could inform future simulation educational practice.

This study set out to provide a deep understanding of students lived experience of emotions as they emerge within a complex simulation, and their perceived impact on students learning. Critical to this research aim was the need to explore emotions as they were experienced *tacitly* during simulation.

## Methods

### Conceptual orientation

Hermeneutic phenomenology sets out to develop valid knowledge by explaining lived experiences and the contextual forces that influence these experiences [25]. Therefore, hermeneutic phenomenology fits conceptually with our study by providing a deep exploration of such lived experiences, which can often lie beneath surface awareness in simulation-based exercises. Our analysis was also sensitised by Merleau-Ponty’s work on embodiment and lived experiences [26]. Not just focusing on objective bodily experiences, but also illuminating perceptual, intentional and embodied dimensions of individual’s experience.

### Setting and context

The study was carried out at Universidad Católica del Norte (UCN), Chile, where the curriculum of medical degree program is competency-based and it lasts 7 years. Students have clinical and simulation-based learning activities throughout the program. In their final years, medical students are expected to attend an emergency WRS learning activity [7].

### Recruitment and sampling

All medical students (n = 56) in their sixth year of training were invited by email to participate in the study. A matrix of willing subjects and their demographic characteristics were used to select a maximal variation sample of subjects to ensure they represented the year group in terms of gender, age and academic achievements (Table 1). Phenomenological research typically recruits small numbers in order to strike a balance between the far-reaching insights gained by broad sampling and the deep understanding of the phenomenon that can be yielded from in-depth analysis. Therefore, we aimed to recruit up to 8 participants. Personal information (pseudo names, age and gender) are not from actual participants. Any resemblance to real person is coincidence.

**Table 1:** Demographic characteristics of participants

Pseudonym	Gender	Age(years)	Grades during internships (1–7)
Mark	Male	23	6.8
John	Male	23	6.2
Mary	Female	23	6.8
Peter	Male	24	5.8
Ann	Female	27	5.5
Alex	Male	24	5.1
Carol	Female	24	5.2
Susan	Female	23	6.4

### Description of the WRS learning activity

Participants were asked to take part in an emergency WRS activity. Participants assumed the role of a junior doctor and were instructed to manage the care of a number of *patients* in a ward round context [7]. Prior to the simulation, participants received a briefing to receive instructions about the task ahead. They were instructed to conduct a ward-round where they had to assess 3 patients and re-evaluate their management plans. During the course of the WRS, they also had to interact with other healthcare professionals, patient relatives and deal with multiple competing tasks and interruptions. The 3 patient cases were chosen given the relevance to the stage of their training: (1) an anxious patient requiring pain reliever following an acute myocardial infarction; (2) family conflict regarding an end-of-life decision; and (3) an unconscious patient brought to the emergency room by her daughter with limited information about their presenting history. Simulated patients (SPs), simulated relatives and healthcare professional confederates (i.e. actual doctors and nurses) role-played in the simulation. All SPs and simulated healthcare professionals were provided with a script to guide their roles and actions. SPs were professional actors. They received 6 h training from a medical doctor who designed the scenarios. Simulated healthcare professionals were actual health care providers who played their own roles following training. The main objectives for the simulation were that students were able to prioritise which case to deal with, make a diagnosis and treatment plan, work in a team and communicate effectively. During the briefing, the session’s objectives were established as well as a fiction contract, confidential agreement, and tending to psychological safety of participants. Simulations typically lasted 25 min. Following the simulation, debriefing occurred using the ‘debriefing with good judgment' approach [27]. SPs were not involved in debriefing.

### Data capture

**Fig. 1:**
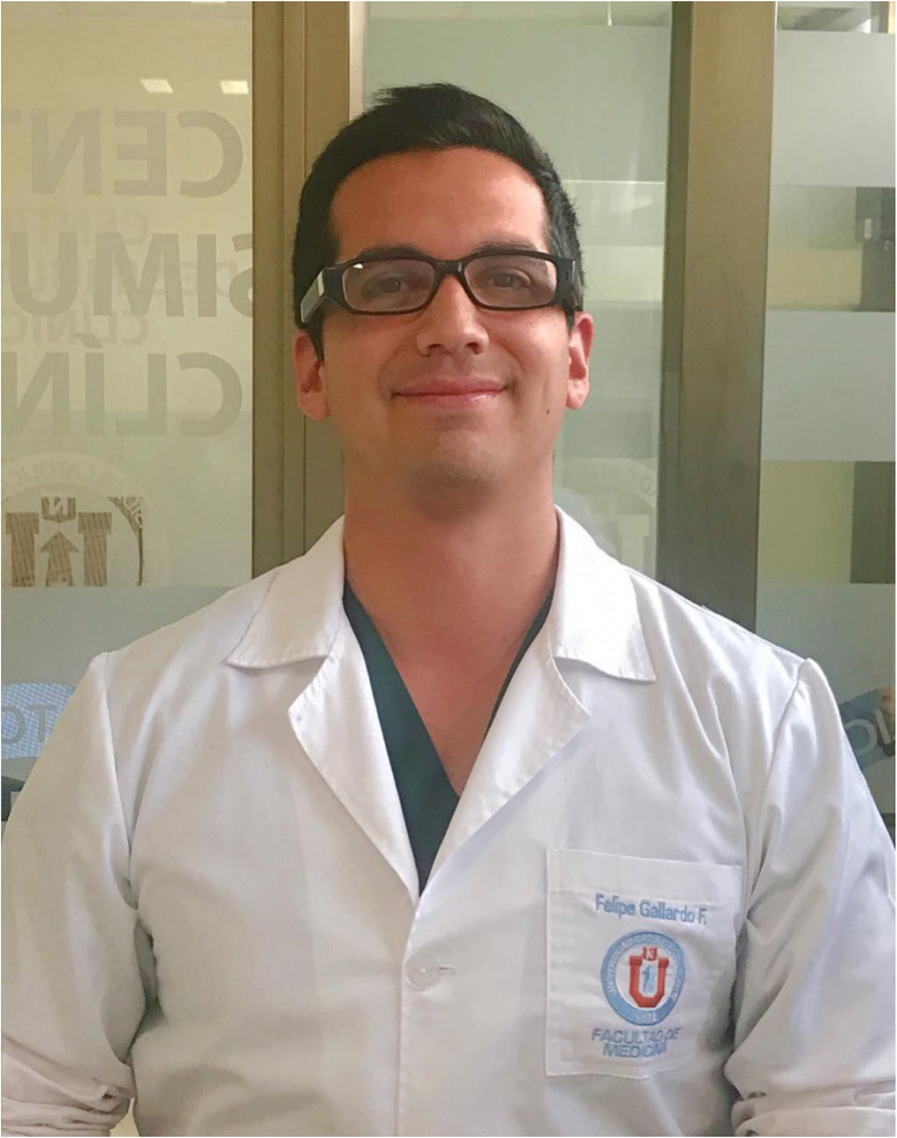
Illustration of a medical student wearing video-glasses

In order to enhance data gathering, participants wore video-glasses (SunnyCam™ HD) to capture, in a first-person perspective, video footage of their WRS experience (Fig. 1).

We captured their entire experience, i.e. video footage *prior, during* and *after* the WRS. Immediately following their WRS activity, participants were interviewed individually by one researcher (CB). Interviews were exploratory and minimally structured to allow discussions to remain as true as possible to their experiences. Footage from participants’ video-glasses was worn to elicit the research interviews in order to remain rooted in their *tacit* experiences of the WRS. Such an interview elicitation method affords interviewers with deeper insights, as they can observe and empathise with what a subject was actually seeing during a particular phenomena [28, 29]. Participants viewed their point of view (PoV) video footage, pausing/starting the video and speaking aloud their experiences, emotions and actions. Participants not only largely led the interview but also the interviewer could pause the video and explore participants’ emotional experiences in a deeper way.

Eight participants took part in the study generating 208 min of video footage and 360 min of interview data. All interviews were transcribed verbatim, checked for accuracy and anonymised using pseudonyms.

### Analysis

We used template analysis to analyse data, because it epistemologically aligned to the hermeneutic phenomenological approach used in this study [30]. Such a form of analysis affords a structured approach to researcher’s interpretation of participants’ lived experiences and how this contributes to the wider understanding of emotions in a simulation-based learning activity.

Prior to conducting the analysis, the researchers brought to the surface their assumptions and presuppositions about the topic. Guided by our research aim, tentative a priori *codes* were created and applied to two initial transcripts. A priori codes were modified in response to analysing the transcripts. Emergent codes were clustered to identify *preliminary themes* and used to develop an *initial research template*. This initial template was then applied to the remaining transcripts and iteratively refined. Finally, all transcripts were coded against the definitive template in order to illuminate the interpretation of the entire dataset. Analysis ended when there was consensus among all of the researchers that a thick and rich description had been achieved. The research team met regularly to discuss the analysis and were continually reflexive.

### Ethics

Ethical approval was given by the Ethical Committee from Universidad Católica del Norte (F.M. 33-2019). A written informed consent was obtained from all participants.

## Results

Analysis yielded four main themes: ‘nervous anticipation’, ‘shock and awe’, ‘in the moment: Flowing and buffeting with the emotions’ and ‘safe landing?’.

### Nervous anticipation

In the time period leading up to the actual simulation, a range of particular emotions were experienced by participants. Emotions could be evoked even prior to arriving in the simulation venue.

“I felt anxious…the weekend before the simulation, I could not sleep while thinking about the simulation…I had A stomachache before I came here, I felt bad.” [John]

In this preparatory phase, *anticipation* dominated participant’s emotional experiences. The emotion of anticipation was often held in tension between being a negative and positive experience. It appeared greater propensity to have a more negative experience, being associated with emotions such as fear and anxiety.

“I felt a lot of anxiety, I wanted to know what kind of patients I would have to attend and if I was going to be able to handle them or not… for me stress and anxiety stimulate my learning if it is not excessive, for example increases my ability to concentrate” [Peter]

Central to these emotional experiences, where participants’ perception of *uncertainty*; uncertainty about *what* they were about to experience and uncertainty of *how* well they would perform. Such experiences triggered with some participants to question their professional ability, both from their *own* perspective and what might *others* perceive (i.e. fellow students and faculty). Often, they experienced a mismatch between their perceived ability and the expected by the faculty. However, such experiences, including the fear of *failing*, had potential to promote participants to concentrate on their learning and perform to the best of their ability.

“I felt anxiety in front of the unknown and I clearly believe that this emotion is super positive for my learning as it predisposes my mind to receive information, process it and make decisions.” [Alex]

### Shock and awe

Moving from the *pre-simulation* to the *intra-simulation* phase marked an important transition in the emotions experienced by participants. As they entered the simulation-suite from the briefing area, an array of intense emotions was induced. Often participants felt overwhelmed with the multiple stimuli and cues they encountered, ranging from the presence of various individuals, clinical equipment, noises and various artefacts. Participants’ point of view video footage would often swing rapidly from side to side (reflecting their head movements) as they started to scope the simulation environment. Participants often felt emotions of anxiousness, describing features of stress and hypervigilance. Such visceral and intense experiences had potential to hinder any learning at that time. The vastness of possibilities, and wide ranging sensory stimuli, often mediated participants to feel overwhelmed; leaving them grappling to make sense of what lay ahead for them in the WRS.

“When I started, I felt impacted, surprised, it was too much…. I did not know who was more serious than the other…” [Mark]

Such intense negative emotions began to reduce when participants started to assimilate and orientate themselves to the scenario. In doing so, participants began to consider where best to focus their attention. Engaging with key individuals in the simulation enabled them to direct their efforts and accommodate more to the situation; selectively orienting themselves to key materials (e.g. clinical observation changes) and social (e.g. nursing staff) dynamics within the simulation. Attuning to the complex dynamics within the simulation gave participants a greater sense of control in their learning and reducing their negative emotions.

“When I entered, I felt initially disoriented, I saw so much to do and did not know how to start, but once the time passed I began to feel a little more confident and I felt a little more free that I could do what I thought was right …” [Ann]

### In the moment: flowing and buffeting with the emotions

As the WRS unfolded, participants experienced a diverse range of emotions. Both the contextual demands within the simulation and how participants perceived their performance to meet these demands had potential to mediate their emotional experiences. Whilst more negative emotions were experienced (e.g. fear and anxiety), the simulation could also evoke more positive emotions such as pride, eagerness and satisfaction.

“With the first patient I felt calm because I already had the whole scheme in my head of how to treat it, but when I saw the second patient, and I heard the patient complain, I did not know what to do, I was confused, anxious…” [Alex]

As participants progressed in the WRS, they became more attuned to their own perceived notion of how well, or not, they were performing. They drew upon their self-sense of achievement and also any clues provided to them during the WRS (for example if a patient responded to their treatment). If participants sensed a more positive outcome because of their actions, they felt rewarded. Such a dynamic had potential of promoting more positive emotions and improving their confidence for learning in the next stages of the simulation.

“When I saw the ST segment elevation in the ECG I felt calm… I knew what to do…this give me confidence in my knowledge, in knowing that I am doing the right thing and that the patient improved, I will remember this when I am seeing a real patient with a similar history” [Mark]

If participants perceived their progression in the simulation to be less than satisfactory, this could induce more negative emotional experiences such as frustration and embarrassment. In such occasions, participants experienced feeling more scrutinised and fearful of being judged as incompetent. Experiencing such negative emotions had the potential of negatively affecting their confidence. Even if they subsequently perceived themselves to have had a positive achievement in the WRS, such negative emotions could persist throughout the simulation. Depending on the degree of such negative emotional experiences, participants could be spurred on to draw upon this experience and develop their learning.

“I felt insecure because I did not remember the doses... I felt a little disappointed of myself…. it motivated me to study, then do it again and see how I am improving!” [Mary]

In more extreme circumstances, participants experienced despair. In such situations, they were less likely to recall or transfer their knowledge into actions. Participants could experience being *paralysed in-the-moment*, which hindered their learning. This could create a negative-loop through affecting their subsequent performance in the WRS. If this occurred, participants tried to suppress any outward signs of these negative emotions to faculty and others. However, internally these negative emotions were prominent.

“…Even after examining her, I felt confused, I did not know what she had and I did not know what to do.... but at that moment, this was negative for my performance, I was as blocked, my mind went blank, I did not know what else to do and I just keeping trying to keep going.” [Susan]

### Safe landing?

Following the simulation, participants often experienced a change in their emotional state. In this *post-simulation* phase, participants transitioned from ‘performing’ to now ‘being’ a student wanting to develop his/her learning. Often, participants experienced relief that the simulation had concluded. As participants assimilated their actions, they began to judge if they had performed well or not. If they felt that they had performed well, participants would experience more positive emotions such as pride, joy and satisfaction. Such emotions promoted their self-esteem and confidence in their learning. Equally the simulation debrief would further enhance these positive emotions and promote their learning.

“I felt joy, pride in myself, of doing things well because I feel that I have worked so hard to be here … during debriefing I could reaffirm that I know, that I have the knowledge and skills to face real patients in the near future. I felt much more positive emotions that reaffirm me that I know and that promote my learning” [John]

When participants perceived that their performance was suboptimal, this could reinforce and evoke further negative emotions such as frustration and shame. The debriefing had potential to reduce such negative emotions and promote learning.

When the simulation finished I felt relief, I had a little stress. When you showed me that I had failed to examine the patients, I felt shame and guilt because it is something that I should have done well… In the debriefing I felt confident, calm and I took a lot of positive things for improving [Carol]

On occasions, if the negative emotions were intense, this could hinder any sense of learning, even despite the best efforts of the debrief process.

## Discussion

By using PoV video footage, we were able to bring to the surface students tacit emotional experiences, i.e. ‘eliciting the tacit’ [24]. The study results indicate that complex simulation can evoke intense positive and negative emotions in students, which are closely linked to their perception of how well they have performed in the simulation. As emotions emerged during the complex simulation, they appear to be highly contextual.

We used simulation to support medical students’ professional skills development. However, our participants perceived simulation as more a *judgemental,* than as a *supportive* or *learning* experience. This tension of feeling judged was experienced throughout the entire simulation and was perceived by students to influence their emotions and learning. The perceived judgemental intent of simulation by learners has been echoed by other researcher [27, 31].

Evident from our findings was that students had a continual desire to gain a sense of how well they were progressing. Students would strive to seek information from their external environment of how well, or not, they were making progress. Whether clues from faculty or the impact that their decisions had on simulated patient care. How students perceived their degree of progress was tightly bound to their emotional experiences. If students experienced a positive progression, this could evoke more positive emotions such as pride and joy. This emotional dynamic could instil more pleasure within student’s experiences and act as an enabler in their performance within the simulation. However, if students sensed that they were not making progress, this could induce more negative emotions and displeasure for students. Our findings showed that such negative emotions could persist despite the simulation context moving on. Such an experience could have a potential detrimental effect on their learning giving that they start questioning their competence, which is consistent with achievement emotions theory.

Integral to student emotional experiences was a sense of professional identity. As the simulation progressed, students often toggled between the notion of being a *medical student* carrying out a simulation to being a *health care professional* of the future. This could imbue a sense of their ability, or inability, of becoming a competent future doctor.

In understanding the interplay between emotions and professional identity, drawing upon socio cultural theory could provide some insights. If we consider the work of Goffman ‘The Presentation of Self in Everyday Life’, he theorised that during social interactions, individuals utilised means in an attempt to exert control over the perceptions of others about their identity [32]. This aligns with what students experience in complex simulation [33]. Possibly being labelled as having a skill deficit, such as not being competent within a simulation scenario, was experienced as a discrediting process that made them doubt about their future performance and competence. In such situations, students made efforts to ‘bottle-up’ their emotions. It could be argued that by concealing their true emotional experiences was an attempt for them not to be judged or labelled as being incompetent.

Learners experienced a diverse array of emotions throughout the entire simulation process, including the phase prior to and after the simulation scenario. Interestingly, specific emotions can be evoked in learners even prior to attending the physical environment of the simulation venue. From the point of view of achievement emotions theory, such a finding is important when considering how best we manage and attend to the emotional experiences of students as they approach a simulation.

### Lines of action in shaping a supportive emotional experience in simulation

As a result of this research, there are a number of potential implications for practice.

Firstly, it is important to increase the awareness of the intense emotions that can be experienced by learners in more complex forms of simulation. Importantly, such emotions are not just heightened within the simulation scenario, but throughout the entire simulation process—even prior to attending the simulation centre. By allowing faculty and supporting teaching staff, such as simulated patients, to be more aware of these emotions, could allow them to be more critically reflective of how best to design and deliver simulation-based educational activities. This is of particular importance when students may exert efforts to conceal their true emotional experiences. By recognising such intense emotions experienced by learners may allow faculty to modify aspects of the simulation were possible in order to best optimise learning, for example reducing the number of distractions and interruptions that a student might receive. This action may help to reduce extraneous cognitive load and free up space in working memory to enhance learning.

Secondly, our results further reinforce the importance conveying a more *supportive* rather than a *judgemental* intent to simulation. Such a notion is pivotal on the emotions experienced by students in simulation. Students are highly sensitive to any evaluatory properties that they can experience from the simulation. Therefore, by setting an intent of support rather than judgement throughout the *entire* simulation process is of critical importance, particularly in the briefing process. Ultimately, there is a call to further enhance a culture of respect and openness in simulation. Very often in the health care area, there can be a culture of blame and defensiveness in admitting error or suboptimal practice [34]. We should strive to promote a greater supportive intent in simulation for learners and a culture that it is safe to take risks without fear of embarrassment. Central to this process is to help students overcome a desire to suppress negative emotions. By promoting students to attune and accept their emotions is an important first step. As we found in our research, at times learners’ emotions were intense and potentially overwhelming. If this occurs, taking time out or pausing the simulation may allow a student to adapt to their emotional state and the simulation.

Lastly, there is an argument to adapt or personalise simulation to students’ emotional states. Despite simulation being a constructed form of experiential learning, it is not experienced by students in a uniform fashion. The emotions experienced in simulation are very much personal, individual and difficult to predict. As students work to the edge of their abilities, there is a fine tipping point of potentially overwhelming students emotionally. By attuning to students emotional states and dynamically modifying the intensity of simulation may provide a more emotionally conducive learning experience for students. How faculty could adapt simulation to learners’ emotional experiences is worthy of future research. In addition, exploring how SPs experience learner’s emotions, and their reactions to these emotional cues, would also be worth exploring.

## Limitations

The results of this study have to be considered alongside its limitations. Given the conceptual orientation in this study, generalisability was not an intended aim. Simulation-based educational activities are diverse in terms of their characteristics and challenges placed on learners. In our study, we considered a more complex form of simulation that extended medical students to the edge of their abilities. Therefore, our findings may not be transferable to all forms of simulation-based education. In future studies, mapping out medical students lived experiences in other forms of simulation would be worthy of research.

Simulation evoked particular emotional experiences throughout the entire process. However, we did not explore the emotional experiences after the simulation process concluded. Exploring students’ emotional experiences beyond the simulation venue would be worthy of future research.

## Conclusions

Complex simulation can evoke complex and intense positive and negative emotions in learners, which can support or hinder learning. Such emotional experiences are tightly bound to the context of the stimulation and how students perceive their performance. Despite the controlled nature of simulation-based education, student’s emotional states are dynamic, often unpredictable and at times fragile. Our findings provide a foundation to guide educators to anticipate and adapt scenarios for managing the *emotional climate* within simulation in order to optimise an emotional *sweet-spot* for learning.

## Data Availability

The datasets used and/or analysed during the current study are available from the corresponding author on reasonable request. Erik Driessen, PhD, is a Professor of Medical Education at the Department of Education and Research, Faculty of Health Medicine and Life sciences, Maastricht University in the Netherlands. His area of expertise lies in learning and assessment in the workplace. https://orcid.org/0000-0001-8115-261X Diana Dolmans, PhD, is a Professor at the School of Health Professions Education (SHE) Maastricht University in The Netherlands. Her special interest relates to teaching and learning in innovative learning environments. Gerard Gormley, M.D, PhD, is a Professor in the Centre for Medical Education, Queen’s University, Belfast, Northern Ireland. He has a special interest in complexity in simulation-based learning and social cultural processes in summative clinical assessments.

## Abbreviations

WRS: Ward round simulation
UCN: Universidad Católica del Norte
PoV: Point of view
